# Metachronous, Single Metastasis to the Parotid, from Primary Breast Cancer: A Case Report and Review of the Literature

**DOI:** 10.1155/2016/3965283

**Published:** 2016-01-28

**Authors:** Michel Kmeid, François G. Kamar, Selim Nasser, Nabil Moukarzel

**Affiliations:** ^1^School of Medicine, Lebanese University, Beirut, Lebanon; ^2^Department of Hematology-Oncology, Clemenceau Medical Center, Beirut, Lebanon; ^3^Department of Pathology, School of Medicine, Lebanese American University, Byblos, Lebanon; ^4^Department of Otolaryngology, Head and Neck Surgery, School of Medicine, Lebanese University, Beirut, Lebanon

## Abstract

*Background*. The parotid gland is an unusual site for metastatic disease and when metastasis occurs, it commonly originates from head and neck primaries. Spread from distant infraclavicular sites such as the breast, into the parotid, is even more unusual with very few cases reported in the literature.* Case Report*. We describe the case of a 65-year-old woman presenting for a rapidly enlarging right parotid mass. She had a history of an invasive ductal carcinoma of the right breast and was disease-free in the past 6 years prior to her presentation. She was thereafter diagnosed as having a solitary parotid metastasis from breast origin. A total parotidectomy was done and she was referred for adjuvant radiotherapy.* Conclusion*. Any parotid metastasis should be investigated, especially in patients with a prior history of cancer where the possibility of metastasis, even if improbable, should be kept in mind. Fine needle aspiration biopsy (FNAB) is the first diagnostic procedure to be done and immunocytochemistry can provide valuable information even if it is not always needed for diagnosis. Superficial parotidectomy when feasible with adjuvant radiotherapy is the preferred approach for solitary metastasis of the parotid. The prognosis, however, remains poor regardless of the treatment modality used.

## 1. Introduction

Malignant neoplasms of the salivary glands are uncommon constituting approximately 3–5% of all head and neck malignancies and 0.5–1% of all cancers [1]. The incidence of malignant salivary gland tumors is estimated at 1.3 cases per 100000 per year [2] accounting for about 12% of cases of cancer of the oral cavity and pharynx [2], the latter representing 3.5% of all cancers in men and 1.5% of all cancers in women [3]. Primary malignancies of the salivary glands are by far the most frequent, and although metastatic disease to the salivary glands represents less than 10% of salivary gland tumors [1], it should be considered on the list of differentials. The rich lymphoid content of the salivary glands makes an ideal ground for seeding of head and neck primaries. Malignancies from distant infraclavicular sites also spread to the salivary glands, usually to the submandibular glands and to a lesser extent the parotids. The spread is most likely hematogenous [4]. Although rarely encountered, metastatic disease to the salivary glands is widely reported in the medical literature as isolated case reports or small series. In the parotids, metastases from breast, kidney, and lung primaries are the most frequently reported ones. We are describing herein the case of a postmenopausal patient who presented with a cheek mass associated with a partial peripheral facial nerve palsy, caused by a parotid gland metastasis from breast origin, with a review of the literature.

## 2. Case Report

A 65-year-old lady from Syrian descent was referred to our institution in September 2014 for painless rapidly enlarging mass of the right cheek, which she noted only for 2 months prior to presentation. Relevant history included an early stage right breast invasive ductal carcinoma diagnosed 6 years earlier and treated by a lumpectomy with ipsilateral axillary lymph nodes dissection, followed by 6 cycles of adjuvant chemotherapy with CMF (cyclophosphamide, methotrexate, and 5-fluorouracil), focal radiotherapy to the chest wall and right axilla, and subsequent hormonal manipulation with letrozole for 5 years.

The patient was disease-free for the following 6 years until a growing right cheek mass led her to seek medical advice.

Physical exam revealed a palpable firm, nontender, nonmobile right inferior parotid mass of 2 cm at the level of the angle of the mandible. The right corner of the mouth dropped with asymmetry that increased with facial expression, thus indicating right mandibular branch palsy. The rest of the exam did not reveal any palpable cervical, supraclavicular, or axillary lymph nodes on both sides. Fibroscopy was done and was normal.

**Figure 1:**
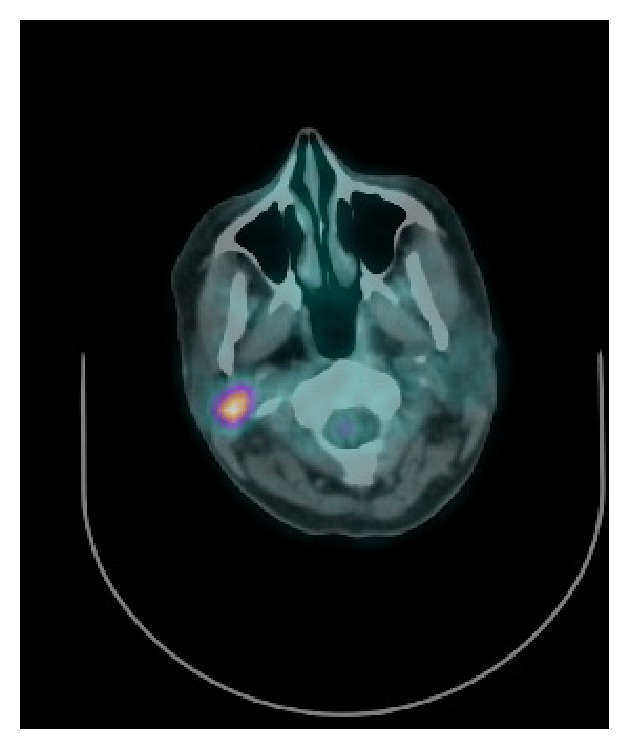
Pet CT showing accumulation of 18-FDG in the right parotid gland.

Contrast enhanced MRI scan of the neck revealed the presence of a 2 cm ill-defined mass of the right parotid gland at the inferior border, hypointense on T1 and mild hyperintense on T2 with moderate enhancement after gadolinium injection. No cervical lymphadenopathy was detected. Fine needle aspiration biopsy (FNAB) of the parotid mass revealed the presence of malignant cells of breast origin. Staging FDG-PET CT scan was obtained showing accumulation of 18-FDG in the right parotid and ruled out other distant or locoregional metastases (Figure 1).

**Figure 2:**
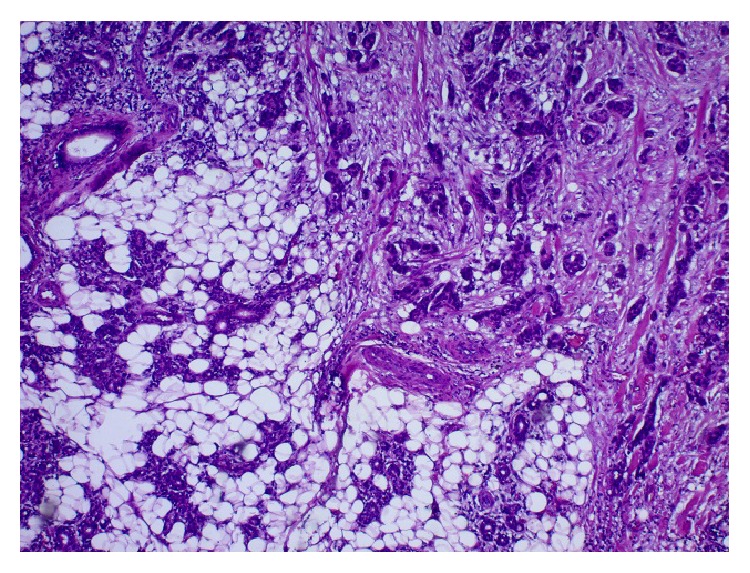
Parotid: H&E ×40: neoplastic ducts (right upper part of the image) invading the parotid parenchyma (left lower part of the image).

**Figure 3:**
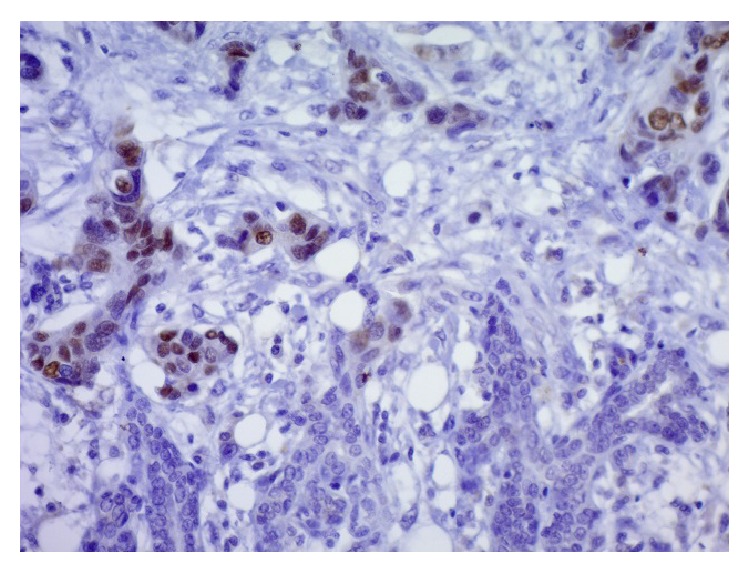
Parotid: immunostain with anti-estrogen receptors antibody ×40: the nuclei of the malignant cell are highlighted in brown. Parotid ducts are seen in the lower part of the image.

**Figure 4:**
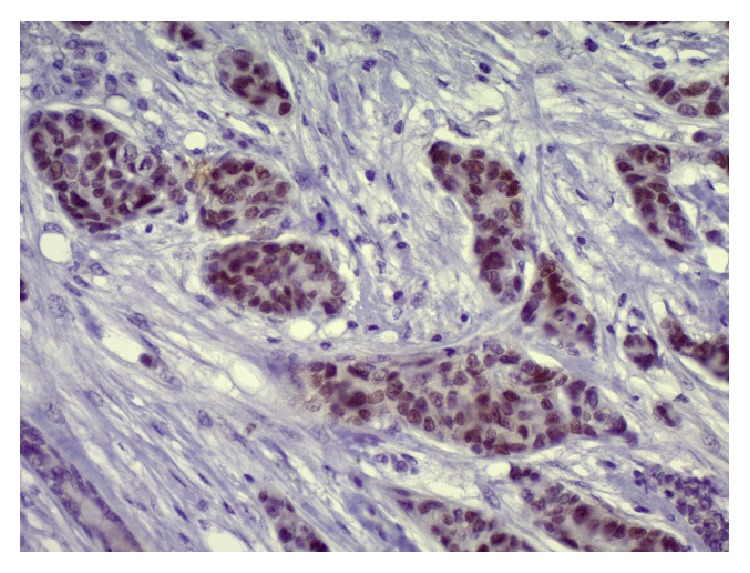
GATA3 nuclear expression in neoplastic cells (GATA3 immunostaining ×200) confirming the breast origin of the parotid tumor.

A total parotidectomy was subsequently done with preservation of all facial nerve branches. However, the dissection of the mandibular branch was done with probable remnant of tumor cells on the nerve. The retroauricular vein was sacrificed because of direct invasion by the tumor. The anatomic pathology report confirmed the secondary nature of the tumor (of breast origin) invading the parotid parenchyma with perineural and perivascular invasion (Figure 2). Although immunocytochemistry was not done on the FNA sample and was not needed for diagnosis, immunostaining with anti-estrogen receptor (ER) antibodies was performed on the final surgical pathology specimen and further supported the diagnosis as the tumoral cells were ER positive (Figure 3). GATA3 immunostaining, a specific marker for breast cancer, was also done confirming the breast origin of the tumor (Figure 4).

The patient tolerated well the procedure and was referred for adjuvant radiotherapy.

## 3. Discussion

Parotid metastasis of breast origin is an extremely rare event, and only few cases are reported in the literature.

The parotid glands are the largest major salivary glands and are the only salivary glands to contain intraglandular lymph nodes; the submandibular and sublingual glands do not. The parotid lymph nodes are divided into 2 layers: a superficial nodal layer located between the gland and its capsule and a deep layer located within and beneath the gland [5]. These lymph nodes drain the frontal and lateral aspects of the scalp, lateral aspects of the lids, conjunctiva, external auditory canal, root of the nose, the lacrimal gland, and the parotid itself.

Metastatic disease involving the parotid accounts for approximately 9 to 14% of all parotid tumors [6]. Head and neck tumors make up to two-thirds of these metastases, squamous cell carcinoma and malignant melanomas from the upper airway and the foregut being the most common, followed by the skin [7]. However, metastases from distal primaries such as the breast, GI tract, kidneys, and prostate are quite rare and thought to reach the parotids through the thoracic duct or Batson's paraspinal venous plexus, skipping the pulmonary filter [8].

The submandibular glands can also be involved with metastatic spread. However, due to lack of any lymphatic network draining the skin and subcutaneous tissue of the head and neck, 85% of such metastasis arises from infraclavicular primary tumors, most commonly breast, kidney, and small cell lung carcinomas [9]. Squamous cell carcinoma of the head and neck is unlikely to metastasize into the submandibular gland and when it does so, the primary tumor is usually in the ipsilateral oral cavity in close proximity to the gland or it has spread into an adjacent level I cervical lymph node [10]. Therefore, metastasis to the submandibular gland from head and neck primaries occurs primarily by direct contiguous invasion, whereas those originating from distant infraclavicular sites spread via the hematogenous route. To our knowledge, metastatic tumors have not been described in sublingual or accessory salivary glands.

Very few cases have been described in which the parotid was the only or major site of metastasis [11]. Parotid metastases are also reported synchronously with the initial presentation of the patient and metachronous years after the primary diagnosis [12] as in our case, after 6 years of being disease-free. Metastases below the clavicle are mostly from renal, lung, and breast carcinoma [13]. Less commonly, metastases from primary tumors of the gastrointestinal or genitourinary tracts into the parotid are reported. And anecdotal cases such as a primary urachal adenocarcinoma of the bladder [14], gastric adenocarcinoma [13], and hepatocellular carcinoma [15] were reported.

The majority of parotid metastasis from breast origin reported in literature is of invasive ductal carcinoma [6]. However, metastases from invasive lobular carcinoma and even malignant phyllodes tumor were described. Cases of involvement of the contralateral parotid are equally reported as the ipsilateral one which further supports the likely hematogenous route for tumoral spread versus direct lymphatic invasion [4].

Most patients with a parotid neoplasm present with a painless mass or swelling of the gland. Signs or symptoms of facial nerve involvement are suggestive of a malignant origin rather than a benign tumor. Up to 30–40% of patients with parotid malignancies present with peripheral facial nerve paralysis [12]. Radiological workup with computed tomography and MRI is used to further support the clinical assessment regarding the benign or malignant nature of the tumor and to define its location (intra- versus extraglandular) and extent and detect any nodal or distant spread. However, one cannot distinguish a primary malignant tumor of the parotid from a metastasis based on imaging criteria [16].

Fine needle aspiration (FNA) has an important role in the workup of a parotid mass as it can assist the surgeon in treatment planning in case of malignant involvement. FNA has an 85% accuracy in distinguishing between malignant and benign lesions of the parotid and can differentiate primary neoplasms of the parotid from metastatic disease [17]. Rarely, FNA results may be misleading such as a case of parotid metastasis from hepatocellular carcinoma reported by Yu et al. where FNA did not show any specific cytopathologic features to allow an appropriate diagnosis.

Regarding spread from breast origin, FNA can pose a diagnostic challenge for the cytologist, as primary salivary duct carcinoma and metastatic ductal carcinoma from the breast share many morphological and immunocytochemical characteristics. This emphasizes the importance of communicating the clinical history to the pathologist as primary salivary duct carcinoma mainly affects elderly males and its association with ductal carcinoma of the breast (metachronous or synchronous tumor) is unlikely and rarely described in the literature [6]. When metastatic carcinoma is identified on parotid FNA, restaging with FDG-PET CT scan is recommended to evaluate locoregional and look for distant disease. Treatment is planned according to radiological staging.

When cytopathology result fits with the clinical history, it is sufficient to make an appropriate diagnosis as reflected in our case. Metastatic ductal carcinoma to the parotid has an infiltrating aspect, often showing residual normal parotid acini between the neoplastic glands. In contrast, salivary ductal carcinoma is infiltrative but also expansile, leaving no or very rare normal parotid gland elements between its neoplastic cells. Moreover, metastatic breast ductal carcinoma lacks the aspect of intraductal cribriform carcinoma that is characteristic of primitive salivary ductal carcinoma of the parotid gland [18]. Immunocytostaining adds little benefit as primary and metastatic ductal carcinoma of the parotid share similar immunocytochemical features; however, the absence of expression of estroprogestinic receptors favors the diagnosis of a primary ductal tumor of the parotid gland [18]. In addition, salivary ductal carcinoma has been reported to express almost invariably androgen receptors [19]. Comparing the hormone receptors immunoprofile of both the parotid tumor and the primary breast tumor can be helpful although phenotypic discrepancies regarding the expression of hormone receptors between primary tumor and metastasis have been described in up to 25% of cases [7].

The management of a sole parotid metastasis of breast origin is still controversial. Nevertheless, an appropriate parotidectomy with negative margins and with preservation of the facial nerve when possible is preferred. Superficial parotidectomy was successful in providing local control in most cases [12]. In our case, the deep lobe of the parotid was involved and total parotidectomy inevitable. Some authors suggested ipsilateral neck dissection [8, 16]; however, only limited data exists regarding the benefit of such procedure, such cases being very rare. Shi et al. advocate the use of an ipsilateral neck dissection when the metastasis is from head and neck primaries as spread occurred predominantly via the lymphatic system, whereas, in cases of hematogenous spread from distant sites, neck dissection is thought to be unnecessary. Adjuvant radiotherapy to the parotid and neck is recommended by most authors for patients without nodal involvement; however, others favor the use of adjuvant chemotherapy, the rationale behind it being the possible coexistence of occult and microscopic metastasis, reserving the use of irradiation for cases where local control could not be achieved by surgery alone [7].

Although metachronous solitary parotid metastases with longer disease-free survival are considered as good prognostic factors [16], many authors consider that parotid surgery does not improve life expectancy [14], and the management of a parotid metastasis is palliative regardless of the therapeutic modality used as the prognosis of such patients is dismal with a 5-year survival rate of only 10% [6]. Our patient underwent a total parotidectomy with preservation of the facial nerve; however, we believe microscopic tumor was left behind around the nerve, and the patient was therefore offered postoperative focal radiotherapy.

## 4. Conclusion

A parotid mass in a patient with a prior history of a malignancy should be investigated and considered as metastasis from the primary disease until proven otherwise. The possibility of coexisting primary neoplasm of the parotid, benign or malignant, should also be investigated and ruled out. FNA is the first step and will guide further diagnostic workup. Immunocytochemistry can provide additional information when histopathologic features are not diagnostic. Superficial parotidectomy and adjuvant radiotherapy are the preferred approach when the parotid is the only site of recurrence. There are no guidelines in this regard and the reported 5-year survival is 10%; treatment is therefore considered by many as palliative.
